# Understanding the functional role of genistein in the bone differentiation in mouse osteoblastic cell line MC3T3-E1 by RNA-seq analysis

**DOI:** 10.1038/s41598-018-21601-9

**Published:** 2018-02-19

**Authors:** Myungsuk Kim, Jisun Lim, Jung-Hee Lee, Kyung-Mi Lee, Suji Kim, Kye Won Park, Chu Won Nho, Yoon Shin Cho

**Affiliations:** 1 Convergence Research Center for Smart Farm Solution, Korea Institute of Science and Technology, Gangneung, 25451 Republic of Korea; 2 Natural Products Research Center, Korea Institute of Science and Technology, Gangneung, 25451 Republic of Korea; 3 Department of Biomedical Science, Hallym University, Chuncheon, 24252 Republic of Korea; 4 Department of Food Science and Biotechnology, Sungkyunkwan University, Suwon, 16419 Republic of Korea

## Abstract

Genistein, a phyto-estrogen, can potentially replace endogenous estrogens in postmenopausal women, but the underlying molecular mechanisms remain incompletely understood. To obtain insight into the effect of genistein on bone differentiation, RNA sequencing (RNA-seq) analysis was used to detect differentially expressed genes (DEGs) in genistein-treated vs. untreated MC3T3-E1 mouse osteoblastic cells. Osteoblastic cell differentiation was monitored by measuring osteoblast differentiation factors (ALP production, bone mineralization, and expression of osteoblast differentiation markers). From RNA-seq analysis, a total of 132 DEGs (including 52 up-regulated and 80 down-regulated genes) were identified in genistein-treated cells (FDR q-value < 0.05 and fold change > 1.5). KEGG pathway and Gene Ontology (GO) enrichment analyses were performed to estimate the biological functions of DEGs and demonstrated that these DEGs were highly enriched in functions related to chemotactic cytokines. The functional relevance of DEGs to genistein-induced osteoblastic cell differentiation was further evaluated by siRNA-mediated knockdown in MC3T3-E1 cells. These siRNA knockdown experiments (of the DEGs validated by real-time qPCR) demonstrated that two up-regulated genes (*Ereg* and *Efcab2*) enhance osteoblastic cell differentiation, while three down-regulated genes (*Hrc*, *Gli*, and *Ifitm5*) suppress the differentiation. These results imply their major functional roles in bone differentiation regulated by genistein.

## Introduction

Bone structure is regulated by a dynamic balance between bone formation mediated by osteoblasts and bone resorption mediated by osteoclasts^1,2^. Osteoblasts, which arise by differentiation from mesenchymal stem cells (MSC), play important roles in osteogenesis^3^, and the continuous differentiation of MSCs causes osteoblasts to undergo maturation and mineralization^3,4^. Bone formation process proceeds through interactions among various proteins^5,6^. For example, osteoblastogenesis is regulated through the activation of crucial transcription factors such as Runt-related transcription factor 2 (RUNX2), along with upregulation of osteogenic proteins including alkaline phosphatase (ALP, an early stage osteoblast differentiation marker), osteopontin (OPN, a mineralization inhibitor), and osteoprotegerin (OPG, an inhibitor of osteoclast differentiation)^7,8^. RUNX2 is itself activated by the BMP signaling pathway, and thus bone morphogenetic protein 2 (BMP2) is a master regulator of osteoblast differentiation^9^. The expression of proteins involved in the regulation of osteoblast differentiation can be influenced by various factors, including growth factors, estrogen, and cytokines^6,10,11^.

Estrogen, one of the sex steroid hormones, is frequently used in hormone therapy because of its importance in maintaining bone mass^12^. Despite its extensive use in treating osteoporosis, estrogen has various side effects, including elevated risks of certain cancers^13^. Thus, the discovery of alternative agents that could replace estrogen would be highly beneficial for skeletal and overall health. Candidates for this purpose include phytoestrogens, plant-derived compounds with structures and functions similar to those of mammalian estrogens. Their use as alternative treatments for osteoporosis, potentially lacking some or all of the side effects of estrogen, has been extensively studied^14^. Genistein is a non-steroidal phytoestrogen found in various plants, including soy, fava, and lupin^15^. Because it is structurally similar to 17β-estradiol, genistein is considered as a potential therapeutic agent for preventing postmenopausal osteoporosis^16^. Genistein inhibits bone loss in part by promoting bone formation^17^, i.e., it contributes to maintenance of bone mass not only by decreasing osteoclastogenesis but also by increasing osteoblastogenesis^18–20^. A previous clinical study reported that intake of soy isoflavone extracts, including genistein, induces stimulation of osteoblast function in postmenopausal women^21^.

Genistein affects several molecular pathways involved in stimulating osteogenic bone formation and inhibiting osteoclastic bone resorption. Genistein induces the expression of ALP, bone sialoprotein (BSP), OCN, and osteopontin (OPN) by activating the p38MAPK–Runx2 pathway, which promotes osteogenesis^22^. Genistein also stimulates proliferation and osteoblastic differentiation through the nitric oxide/cGMP pathway^22,23^. To inhibit osteoclastic bone resorption, genistein acts as an inhibitor of tyrosine kinases^22^. Genistein likely affects the NF-ĸB pathway, which promotes osteoclast formation, via the activities of RANKL (receptor activator of NF-ĸB ligand), RAN (receptor activator of NF-ĸB), and OPG (osteoprotegerin)^22,24^.

Although several molecular mechanisms of genistein have been explored in bone cells, its effect on genome-wide gene regulation involved in bone formation and resorption remains largely unknown. To understand the global molecular mechanisms of genistein in bone differentiation, we conducted RNA sequencing (RNA-seq) analyses in genistein-treated and untreated MC3T3-E1 mouse osteoblastic cells. We also sought to identify novel genes that are associated with the osteogenic responses of MC3T3-E1 cells to genistein treatment.

## Results

### Identification of DEGs in genistein-treated cells by RNA-seq

With the goal of understanding the effect of genistein on bone differentiation, we performed polyadenylated paired-end RNA-seq analysis to identify DEGs in genistein-treated vs. untreated MC3T3-E1 cells. Prior to this experiment, we optimized the dose of genistein for induction of bone differentiation. MTT, ALP, and Alizarin red S staining assays detected the maximum expression of markers of osteoblastic differentiation, without severe cell death, at a genistein concentration of 10 µM (Supplementary Figs 1–3). Based on these findings, 10 µM genistein was administered to MC3T3-E1 cells in both the RNA-seq experiments and subsequent functional studies, described below.

Approximately 311 million paired-end sequencing reads were obtained from six samples (three genistein-treated and three untreated). About 85% of sequencing reads could be mapped to the reference mouse genome using the TopHat and Bowtie software (Supplementary Table 4). Using Cufflinks, mapped reads were assembled to the genome, and genome-wide gene expression levels were determined on the basis of the FPKM normalization. Cuffdiff analysis revealed a total of 132 DEGs (52 up-regulated and 80 down-regulated) in genistein-treated cells that fulfilled our DEG selection criteria, adjusted p-value (reported as q-value in Cuffdiff) <0.05 and fold change >1.5 (Supplementary Table 5 and Supplementary Fig. 4).

**Figure 1:**
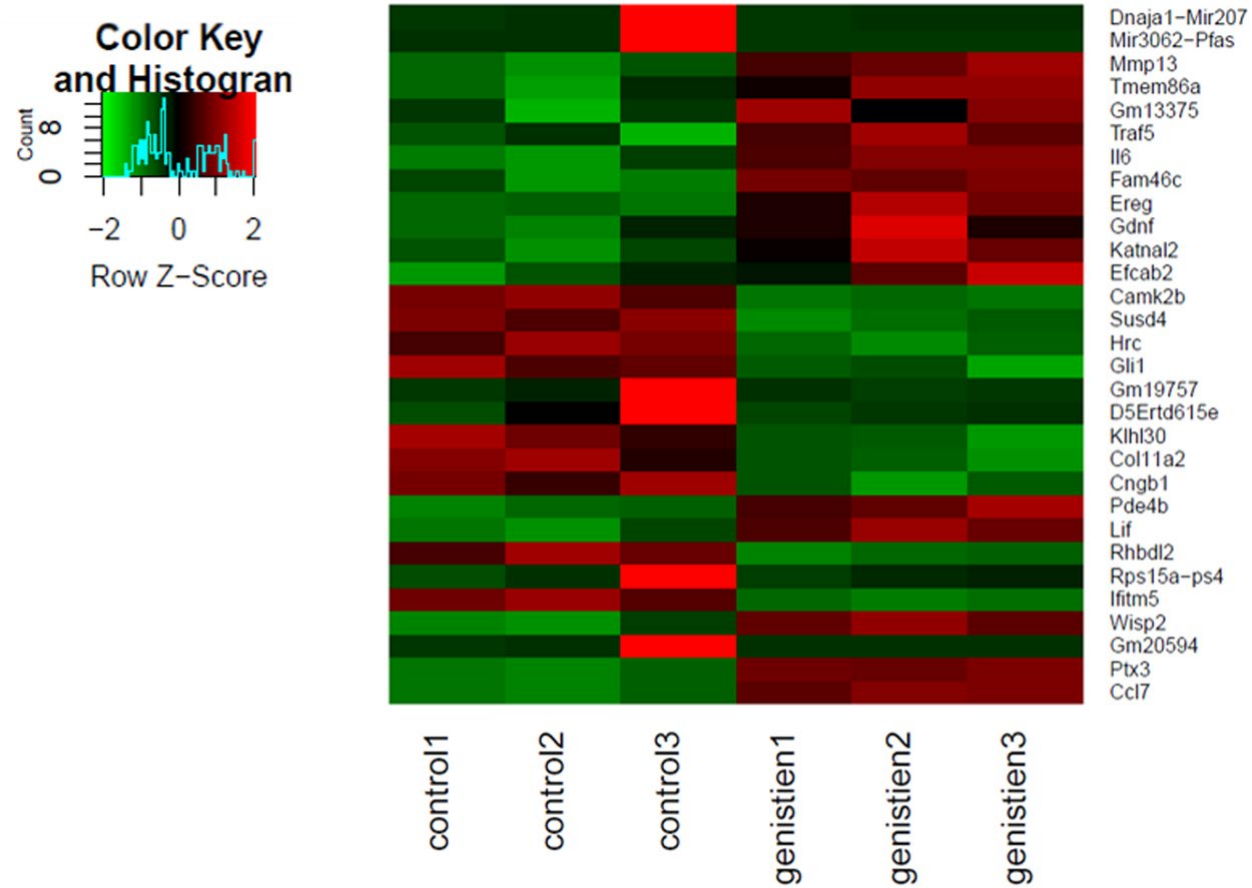
Heatmap of top 15 up- and 15 down-regulated genes in genistein treated mouse osteoblastic cell line MC3T3-E1.

**Figure 2:**
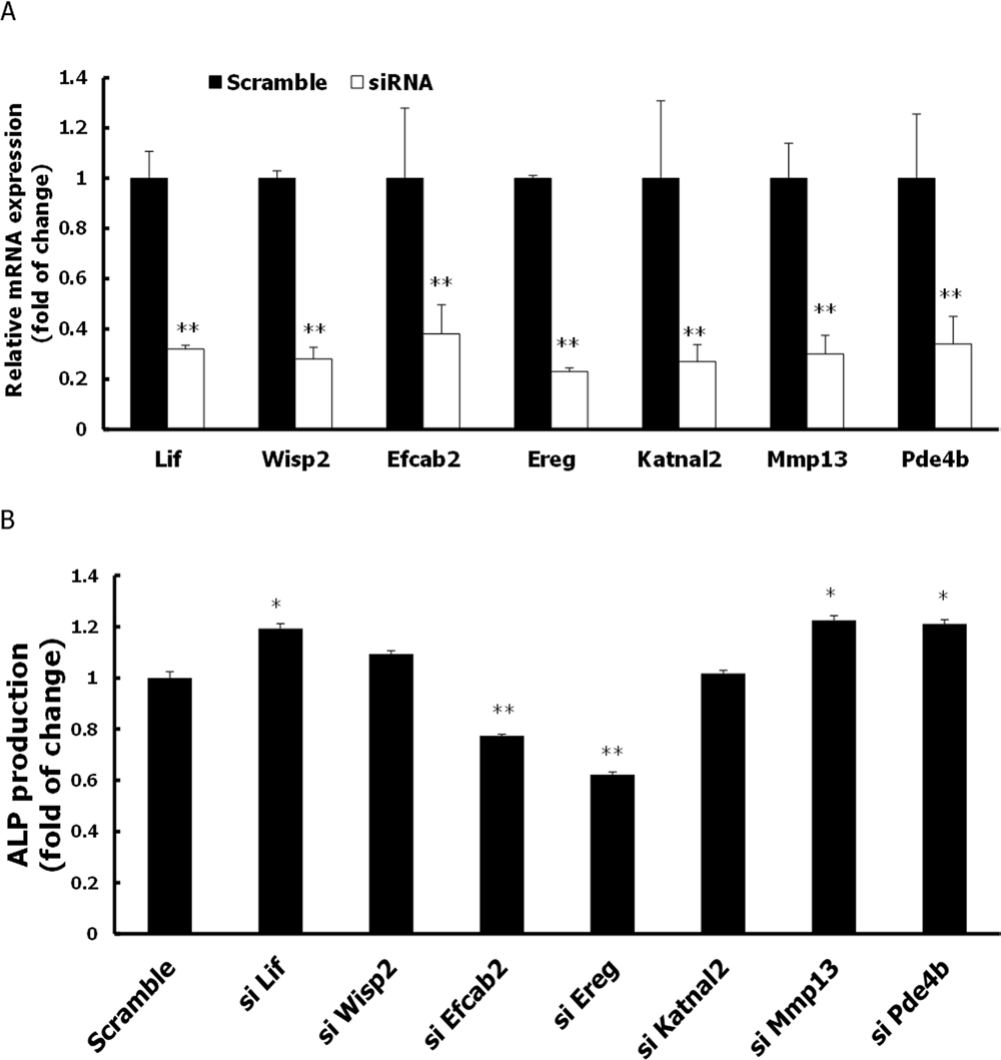
Effect of genistein on mRNA expression of selected differentially expressed genes in MC3T3-E1 cells. (**A**) Analysis of mRNA on up-regulated selected genes (*Ccl7, Lif, Mmp-13, Wisp2, Ereg, Il6, Pde4b, Katnal2, Efcab2, Gdnf*) in RNA sequencing. (**B**) Analysis of mRNA on down-regulated selected genes (*Camk2b, Cxcl9, Gli1, Hrc, Ifitm5, Klhl30, Cngb1, Rhbdl2, Susd4*) in RNA sequencing. Specific mRNA expression values were normalized to the expression of β-actin. Results are expressed as mean ± S.D of three independent experiments. (*P < 0.05, **P < 0.01 compared with control group).

Of 132 DEGs, the 15 up- and 15 down-regulated genes with the largest fold changes in MC3T3-E1 cells (Fig. 1) were selected for the validation by qRT-PCR assays. Of these 30 selected DEGs, we measured the mRNA levels of 20 (11 up-regulated and 9 down-regulated, excluding ten functionally uncharacterized genes) in the presence and absence of genistein. About 70% of qRT-PCR tested genes exhibited expression patterns similar to those observed in RNA-seq experiments (Fig. 2A–B and Table 1). Genistein-regulated expression of these genes was further confirmed in mouse bone marrow-derived osteoblasts (Supplementary Fig. 5).

**Table 1:** Top 11 up- and 9 down-regulated genes among 132 differentially expressed genes (q-value < 0.05 and fold change >1.5) in genistein treated mouse osteoblastic cell line MC3T3-E1. Differentially expressed genes were detected b`y RNA-seq analyses.

Group	Gene Name	Position	log_2_FC	p-value	q-value	qRT-PCR assay
Up-regulated genes	*Katnal2*	18qE3	1.47	2.50E-04	1.65E-02	validated
	*Wisp2*	2qH3	1.17	5.00E-05	4.33E-03	validated
	*Mmp13*	9qA1	1.16	5.00E-05	4.33E-03	validated
	*Pde4b*	4qC6	1.15	5.00E-05	4.33E-03	validated
	*Ereg*	5qE1	1.05	5.00E-05	4.33E-03	validated
	*Efcab2*	1qH4	1.05	6.00E-04	3.18E-02	validated
	*Lif*	11qA1	0.88	5.00E-05	4.33E-03	validated
	*Ccl7*	11qB5	0.99	5.00E-05	4.33E-03	validated
	*Ptx3*	3qE1	0.94	5.00E-05	4.33E-03	validated
	*Il6*	5qB1	0.95	5.00E-05	4.33E-03	not validated
	*Gdnf*	15qA1	0.92	1.50E-04	1.07E-02	not validated
Down-regulated genes	*Camk2b*	11qA1	−1.74	5.00E-05	4.33E-03	validated
	*Gli1*	10qD3	−1.54	4.00E-04	2.33E-02	validated
	*Hrc*	7qB3	−1.48	5.00E-05	4.33E-03	validated
	*Ifitm5*	7qF4	−1.46	5.00E-05	4.33E-03	validated
	*Klhl30*	1qD	−1.43	5.00E-05	4.33E-03	validated
	*Col11a2*	17qB1	−1.50	5.00E-05	4.33E-03	not validated
	*Susd4*	1qH5	−1.39	1.50E-04	1.07E-02	not validated
	*Rhbdl2*	4qD2.2	−1.37	5.00E-05	4.33E-03	not validated
	*Cngb1*	8qC5	−1.35	5.00E-05	4.33E-03	not validated

FC: fold change.

### Determination of biological roles of DEGs by *in silico* methods

To obtain insight into the overall biological effect of genistein on mouse osteoblastic cells, we performed KEGG pathway analysis of the DEGs detected in this study. Of 132 DEGs, 127 were strongly enriched (Benjamini p-value < 0.05) in functions related to the TNF signaling pathway, rheumatoid arthritis, cytokine–cytokine receptor interaction, cAMP signaling pathway, HIF-1 signaling pathway, and amphetamine addiction (Supplementary Table 6). These genes were also significantly enriched (Benjamini p-value < 0.05) in several GO terms. Enriched GO annotations based on biological process (BP) for these genes included cell proliferation, inflammatory response, macrophage chemotaxis, chemokine-mediated signaling pathway, chemotaxis, monocyte chemotaxis, and cellular response to interleukin-1 (Supplementary Table 6). In addition, a number of DEGs were enriched in several GO terms based on molecular function (MF), including growth factor activity, cytokine activity, heparin binding, chemokine activity, and protein homodimerization activity (Supplementary Table 6).

### Functional validation of DEGs by gene knockdown

**Figure 3:**
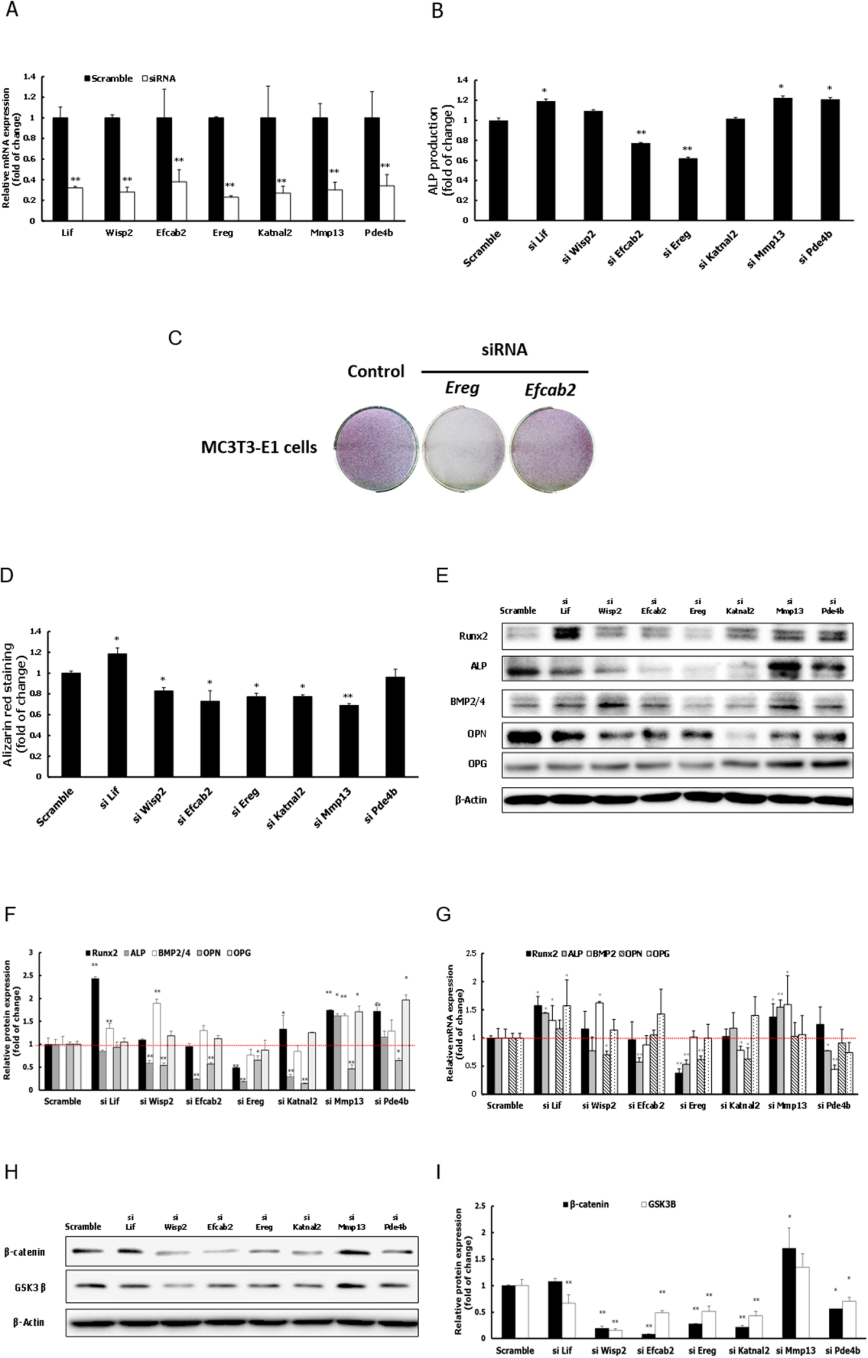
Effect of siRNA-mediated depletion of seven up-regulated genes (*Lif, Wisp2, Efcab2, Ereg, Katnal2, Mmp13*, and *Pde4b*) on osteogenic differentiation of MC3T3-E1 cells. (**A**) The knockdown efficiency of siRNAs targeting seven up-regulated genes compared to scramble siRNA was confirmed by RT-PCR in the presence or absence of osteogenic induction at 3 days after transfection. (**B**) ALP activity after 3 days of osteogenic induction as determined by quantitative ALP activity assay. (**C**) ALP staining after 4 days of osteogenic induction for MC3T3-E1 cells transfected with 40 pmole siRNAs of *Ereg* and *Efcab2*. (**D**) Quantification of Alizarin red S staining after 7 days of osteogenic induction. (**E**,**F**) Protein expression levels of Runx2, ALP, BMP2/4, OPN, and OPG as determined by western blot analysis. (**G**) mRNA expression levels of Runx2, ALP, BMP2, OPN, and OPG as determined by RT-PCR. (**H**,**I**) Protein expression levels of β-catenin and GSK-3β as determined by western blot analysis. Specific protein and mRNA expression values were normalized to the expression of β-actin. Results are expressed as mean ± S.D of three independent experiments. (*P < 0.05, **P < 0.01 compared with scramble group).

To validate the functional relevance of DEGs in bone differentiation, we initially focused on qRT-PCR validated genes (nine up-regulated, five down-regulated) (Table 1). For up-regulated DEGs, we tested seven genes (*Katnal2*, *Wisp2*, *Mmp13*, *Pde4b*, *Ereg*, *Efcab2*, and *Lif*) in siRNA knockdown experiments to evaluate their function in MC3T3-E1 cells. Two other up-regulated genes were not tested because specific siRNAs were not available. Knockdown efficiency of siRNAs in the presence or absence of osteogenic stimulus was assessed by qRT-PCR. mRNA levels were reduced more than 60% by siRNA treatment (Fig. 3A). After osteogenic induction, ALP activity, an early marker of osteoblastic differentiation, was significantly suppressed by siRNA-mediated depletion of *Ereg* and *Efcab2*, but significantly increased by depletion of *Lif*, *Mmp13*, and *Pde4b* (Fig. 3B). Suppressed ALP activity by the reduced levels of expression of *Ereg* and *Efcab2* was visualized by ALP staining (Fig. 3C). This finding was further validated by the observation of ALP staining in siRNA treated M2-10B4 cells that were derived from bone marrow stromal cells from a (C57BL/6J X C3H/HeJ)F1 mouse (Supplementary Fig. 6).

We also assessed extracellular matrix (ECM) mineralization by Alizarin red S staining, another indicator of osteoblastic differentiation in MC3T3-E1 cells. ECM mineralization was significantly inhibited by depletion of five genes, *Ereg*, *Efcab2*, *Katnal2*, *Wisp2*, and *Mmp13* (Fig. 3D). Furthermore, we tested the effect of siRNA-mediated depletion of genes on the expression of osteogenesis-related genes such as *Runx2*, *Alp*, *Bmp2*, *Opn*, and *Opg* in MC3T3-E1 cells (Fig. 3E–G). Among 7 tested genes, knockdown of *Ereg* and *Efcab2* consistently showed the significant reduction of osteogenesis-related markers (Fig. 3).

Wnt/β-catenin signaling plays an essential role in regulation of osteoblast differentiation^25^. To determine whether the effect of up-regulated genes on osteogenesis was mediated by Wnt signaling, we measured protein expression of β-catenin and glycogen synthase kinase 3 beta (GSK-3β) in MC3T3-E1 cells by western blot analysis. The results revealed that expression of β-catenin and GSK-3β was also inhibited by knockdown of *Ereg*, *Efcab2*, *Katnal2*, and *Wisp2* (Fig. 3H–I).

Taken together, these data consistently indicate the functional relevance of two genes, *Ereg* and *Efcab2*, to osteoblast cell differentiation. These results strongly imply that these genes play functional roles in osteogenesis. Furthermore, their osteogenic functions are likely to be mediated, at least in part, by a mechanism involving Wnt signaling.

**Figure 4:**
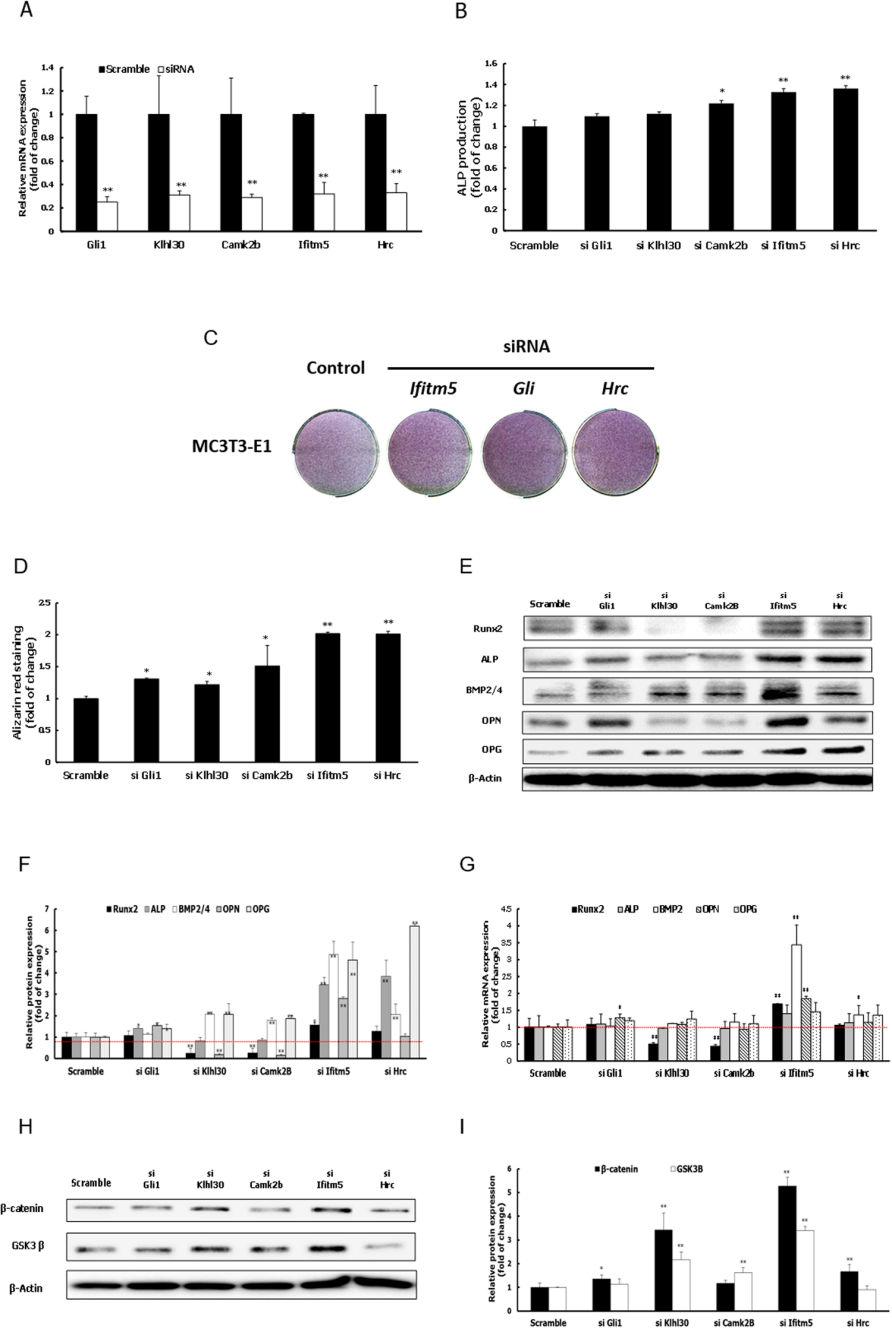
Effect of siRNA-mediated depletion of five down-regulated genes (*Gli1, Klhl30, Camk2b, Ifitm5*, and *Hrc*) on osteogenic differentiation of MC3T3-E1 cells. (**A**) The knockdown efficiency of siRNAs targeting five down-regulated genes compared to scramble siRNA was confirmed by RT-PCR in the presence or absence of osteogenic induction at 3 days after transfection. (**B**) ALP activity after 3 days of osteogenic induction as determined by quantitative ALP activity assay. (**C**) ALP staining after 4 days of osteogenic induction for MC3T3-E1 cells transfected with 40 pmole siRNAs of *Ifitm5*, *Gli1* and *Hrc*. (**D**) Quantification of Alizarin red S staining after 7 days of osteogenic induction. (**E**,**F**) Protein expression levels of Runx2, ALP, BMP2/4, OPN, and OPG in as determined by western blot analysis. (**G**) mRNA expression levels of Runx2, ALP, BMP2, OPN, and OPG as determined by RT-PCR. (**H**,**I**) Protein expression levels of β-catenin and GSK-3β as determined by western blot analysis. Specific protein and mRNA expression values were normalized to the expression of β-actin. Results are expressed as mean ± S.D of three independent experiments. (*P < 0.05, **P < 0.01 compared with scramble group).

To assess the effects of knockdown of the down-regulated genes (including *Camk2b*, *Gli1*, *Hrc*, *Ifitm5*, and *Klhl30*) on osteogenic differentiation, MC3T3-E1 cells were transfected with gene specific siRNAs, and the knockdown efficiency was measured by qRT-PCR. siRNA treatment of MC3T3-E1 cells reduced the mRNA levels of tested genes by more than 60% (Fig. 4A). As expected, ALP activity was increased by depletion of these genes (Fig. 4B). Increased ALP activity by the reduced levels of expression of *Hrc*, *Gli1*, and *Ifitm5* was also visualized by ALP staining (Fig. 4C). This finding was further validated by the observation of ALP staining in siRNA treated M2-10B4 cells (Supplementary Fig. 7).

ECM mineralization was significantly increased by depletion of all tested genes in MC3T3-E1 cells (Fig. 4D). Expression of *Runx2*, *Alp*, *Bmp2*, *Opn*, and *Opg* was also elevated upon depletion of *Ifitm5*, *Hrc*, and *Gli1* following osteogenic induction (Fig. 4E–G). In addition, expression of β-catenin and GSK-3β was increased by knockdown of *Ifitm5*, but not *Hrc* and *Gli1* (Fig. 4H–I). In total, these results indicate that *Hrc*, *Gli1*, and *Ifitm5* suppress osteogenic differentiation of MC3T3-E1 cells. In the case of *Ifitm5*, this suppression may be mediated by the Wnt signaling pathway.

**Figure 5:**
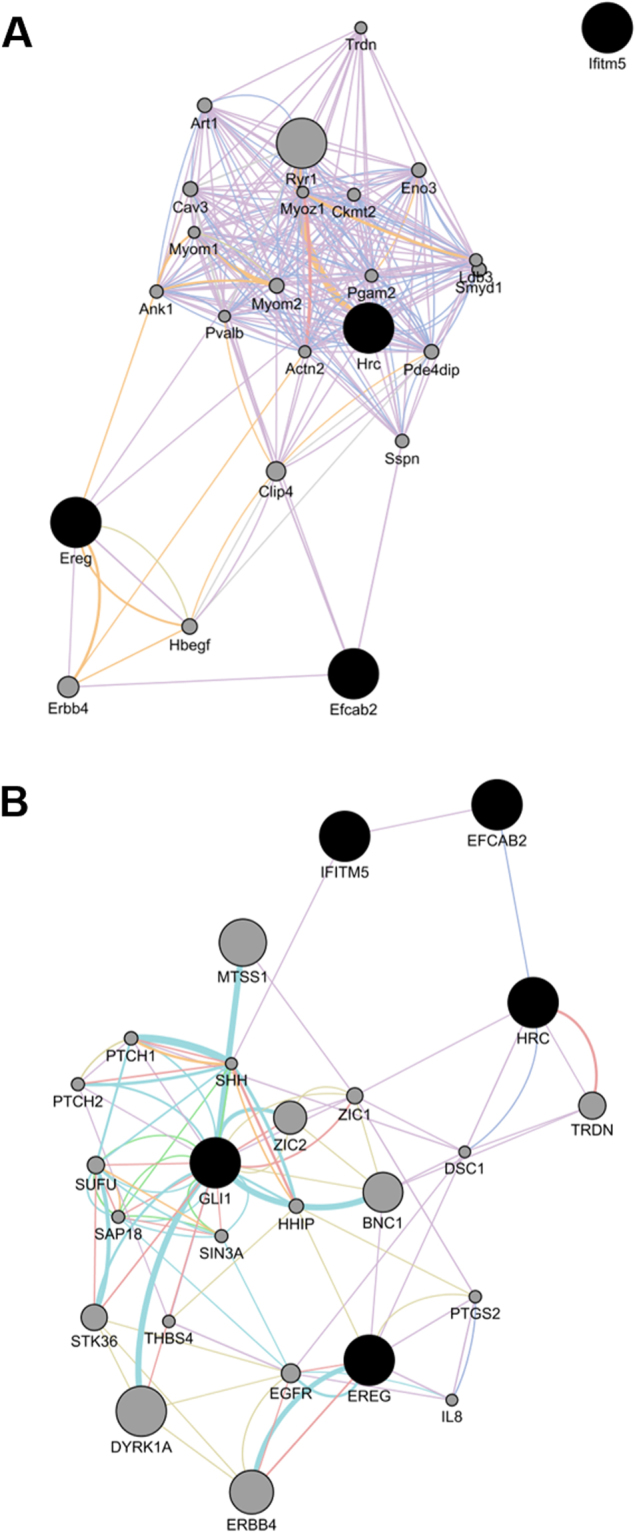
Networks displayed by 3 up- (*Ereg*, *Efcab2*, and *Katnal2*) and 3 down-regulated genes (*Hrc*, *Gli1*, and *Ifitm5*) in genistein treated mouse osteoblastic cell line MC3T3-E1. GeneMANIA software was employed to generate networks to the mouse (**A**) or human (**B**) protein–protein interaction database. Violet, Pink, Orange, Turquoise, Blue, Yellow, and Green lines indicate Co-expression, Physical interactions, Predicted, Pathway, Co-localization, Shared protein domains, and Genetic interactions, respectively.

To obtain insight into the global molecular ensemble regulated by the five crucial genes influencing osteoblast differentiation (*Ereg*, *Efcab2*, *Hrc*, *Gli1*, and *Ifitm5*), we generated networks by applying the GeneMANIA software to the mouse protein–protein interaction database. This analysis revealed one major network comprising Ereg, Hrc and Efcab2 (Fig. 5A). In the human protein–protein interaction database, it is suggested that Efcab2 and Ifitm5 are co-expressed, and Efcab2 and Hrc are co-localized (Fig. 5B).

## Discussion

Genistein is a phytoestrogen, a family of plant-derived compounds that exhibit effects similar to, albeit weaker than, those of mammalian estrogens. Because the MC3T3-E1 cell line expresses estrogen receptors and can differentiate into osteoblasts, it has been a useful model for studying the molecular mechanism of osteogenesis^26^.

RNA-seq analysis is a powerful tool for examining gene expression at the whole genome level. In this study, we identified a total of 132 genes that fulfilled our criteria (q-value < 0.05 and fold change > 1.5) as DEGs (Supplementary Table 5). KEGG pathway analysis demonstrated that these genes have functions related to the TNF signaling pathway, rheumatoid arthritis, cytokine–cytokine receptor interaction, cAMP signaling pathway, HIF-1 signaling pathway, and amphetamine addiction (Supplementary Table 6). In addition, GO biological process and molecular function (GO-BP and GO-MF) annotations for these genes revealed chemotactic cytokines and cell growth as major enriched terms (Supplementary Table 6). Together, these results suggest that genistein induces or inhibits the expression of multiple genes in MC3T3-E1 cells, resulting in regulation of osteoblast proliferation and differentiation.

Among seven up-regulated DEGs tested by siRNA, depletion of two genes, *Ereg* and *Efcab2*, consistently suppressed the maturation potential of MC3T3-E1 pre-osteoblastic cells by inhibiting ALP activity, ECM mineralization, and expression of osteoblast-related biomarkers (e.g., *Runx2*, *Alp*, and *Bmp2*) (Fig. 3). These results strongly suggest that the genistein-induced genes *Ereg* and *Efcab2* may enhance osteoblast differentiation.

*Ereg* encodes a secreted peptide hormone and member of the epidermal growth factor (EGF) family of proteins that can act as a ligand of the epidermal growth factor receptor (EGFR) and the structurally-related Erb-b2 receptor tyrosine kinase 4 (ERBB4). EGFR and its ligands are essential regulators of osteoblast functions and are thus important molecules in bone pathologies^27^. Indeed, EREG protein, an EGFR ligand, promotes cell survival and proliferation, and also increases ALP activity, in primary osteoblasts and MC3T3-E1 cells^28^.

*Efcab2*, which encodes a member of the largest family within the superfamily of calcium-binding EF-hand motif proteins, is a mechano-responsive gene in human osteoblasts^29^. EFCAB2 may regulate bone density by affecting trabecular thickness in a gender-specific manner^30^.

Although previous reports implied that *Ereg* and *Efcab2* play positive roles in regulating osteoblast differentiation and bone formation, no direct evidence confirms the effect of either protein on osteoblast differentiation. In this regard, our findings provide new insight into the genistein-mediated bone differentiation in which these two genes are involved.

In addition to *Ereg* and *Efcab2*, two other genes up-regulated by genistein, *Lif* and *Mmp13*, are also worth attention because of their peculiar expression patterns in bone differentiation. LIF, a pleiotropic cytokine expressed in various tissues, inhibits osteoblast differentiation and induces adipocyte differentiation in fetal rat calvarial cells^31,32^. Although several previous animal studies showed that LIF promotes bone formation^33,34^, a recent study argued that LIF suppresses osteogenesis by inhibiting β-catenin through the LIF/STAT3/SOCS3 signaling pathway^35^. Consistent with its proposed inhibitory function in bone cell differentiation, we found that siRNA-mediated depletion of LIF activated the maturation potential of MC3T3-E1 pre-osteoblastic cells by increasing ALP activity, ECM mineralization, and the expression of osteoblast-related biomarkers (e.g., Runx2, ALP, and BMP2) (Fig. 3). These results supported the inhibitory effect of LIF on osteoblast cell differentiation. Our RNA-seq and qRT-PCR experiments, however, revealed elevated mRNA levels of *Lif* during osteoblast cell differentiation in the presence of genistein. Thus, some unidentified molecular mechanisms may be responsible for genistein-induced bone differentiation, requiring the upregulation of *Lif* in response to genistein treatment to be offset.

Fibrillar collagens, including type I collagen, are major substrates for collagenases such as MMPs, which may regulate osteogenesis by degrading collagens in pre-osteoblastic cells. Among the collagenases, MMP13 is secreted from osteogenic cells and plays an important role in the regulation of osteoblast differentiation^36,37^. In general, a reduction in collagenase activity caused by MMP13 inhibition leads to an increase in the levels of osteoblast differentiation markers, and the reverse is also true when the activation of these MMPs is elevated. MMP13 overexpression results in diminished levels of osteoblastic markers, such as osterix, OPN, and bone sialoprotein, whereas MMP13 knockdown significantly increases osterix and OPN levels and decreases osteonectin levels^38^. As expected from previous findings, we found that siRNA-mediated MMP13 depletion increased the ALP activity and the expression of osteoblastic biomarkers in differentiated MC3T3-E1 cells (Fig. 3). As in the case of *Lif*, however, our RNA-seq and qRT-PCR data revealed upregulation of *Mmp13* during osteogenesis following genistein treatment, suggesting the involvement of unknown molecular mechanisms in genistein-induced bone differentiation. Further studies are required to reconcile this discrepancy.

Among five down-regulated genes tested by siRNA knockdown, depletion of three (*Hrc*, *Gli1*, or *Ifitm5*) significantly activated the maturation potential of MC3T3-E1 pre-osteoblastic cells by increasing ALP activity, ECM mineralization, and expression of osteoblast-related biomarkers (e.g., Runx2, ALP, BMP2, OPN, or OPG) (Fig. 4). This evidence suggests that these three genes may suppress osteoblast differentiation. The role of *Hrc* in bone formation has yet to be fully elucidated. Because its encoded protein is involved in the regulation of calcium sequestration and release in sarcoplasmic reticulum of skeletal and cardiac muscle^39^, we speculate that *Hrc* may suppress bone differentiation by controlling calcium concentrations in osteoblasts.

*Gli1* encodes a member of the Kruppel family of zinc finger proteins that acts as a transcription factor. *Gli1* is activated by the Sonic hedgehog (SHH) signal transduction cascade and regulates stem cell proliferation^40,41^. Acting as a transcriptional activator, GLI1 may regulate the transcription of specific genes during normal development. Thus, considering our findings in this study, we propose that GLI1 regulates several genes involved in the suppression of osteoblast proliferation and differentiation via its role in SHH signaling.

Genome-wide study in cultured osteoblasts demonstrated that *Ifitm5*, which encodes a membrane protein that contributes to bone mineralization, promotes osteoblast differentiation^42^. *Ifitm5* is also expressed particularly in the early stage of osteoblast differentiation^43^. Furthermore, overexpression of *Ifitm5* in osteoblasts promotes bone mineralization, whereas *Ifitm5* knockdown by short hairpin RNA inhibits bone mineralization *in vitro*. Therefore, IFITM5 is considered to be a regulatory factor that promotes mineralization^43^. However, some reports have suggested that this protein also plays an inhibitory role in bone formation. *Ifitm5* knockout mice do not exhibit any abnormality in osteoblast differentiation^44^. Collectively, these results suggest that IFITM5 does not act as a positive regulator of osteoblast differentiation in animal models. In addition, primary osteoblasts isolated from osteogenesis imperfecta type V (OI-V) patients with mutations in *IFITM5* express elevated levels of osteogenesis marker genes such as ALP, bone sialoprotein, OPN, and osteocalcin^45^. These previous reports are consistent with our results from *Ifitm5* siRNA knockdown analyses, supporting its biological function as a suppressor of osteoblast differentiation. Further studies are essential to elucidate the underlying molecular mechanisms responsible for these contradictory findings.

In this study, RNA-seq analysis followed by functional gene classification studies contributed to a holistic understanding of intracellular biological processes. Our RNA-seq analysis detected a total of 132 genes that are directly or indirectly involved in the differentiation of osteoblasts in response to genistein. In MC3T3-E1 cells, genistein treatment altered expression of genes enriched in functions associated with cell proliferation, cell migration, cell differentiation, and inflammatory responses. Subsequent knockdown analyses demonstrated that two up-regulated genes (*Ereg* and *Efcab2*) and three down-regulated genes (*Hrc*, *Gli1*, and *Ifitm5*) play critical roles in the osteoblastic cell differentiation.

Further studies of the roles of the genes identified in this study are necessary to clarify the molecular mechanisms underlying osteoblast differentiation. The generation of stable osteoblastic cell lines lacking or overexpressing specific genes could facilitate the development of therapeutic strategies and the discovery of new bone-related anabolic drugs.

## Materials and Methods

### Cell culture

MC3T3-E1 osteoblast-like cells obtained from the American Type Culture Collection (ATCC, Manassas, VA, USA) were cultured in alpha-minimum essential media (α-MEM) supplemented with 10% fetal bovine serum (FBS) and 100 U/mL penicillin. M2-10B4 bone marrow stromal cells were also obtained from the ATCC. These M2-10B4 cells were maintained in RPMI-1640 containing 10% FBS and antibiotics. Primary bone marrow osteoblasts were obtained by extracting bone marrow cells from mouse femurs. Briefly, individual calvarias was surgically isolated from the femurs were segregated and adherent tissue material was cleaned by gentle scrapping. The pooled calvarias were kept for repeated digestion (15 min/digestion) with 0.05% trypsin and 0.1% collagenase P to release cells. Cells were cultured in a modified essential medium (α-MEM) containing 10% fetal bovine serum (FBS) and 1% penicillin/streptomycin. For induction of osteoblastogenesis, the cells were incubated in differentiation media (50 µg/ml ascorbic acid and 5 mM β-glycerophosphate). Cells were grown at 37 °C in a 5% CO2 atmosphere. Cell culture medium was changed every 3 days.

### Animal

Female C57BL/6 J mice (18 ± 5 g) were obtained from Central Laboratory Animal Inc. (Seoul, Korea). Animals were kept in a 12-h light–dark cycle, with controlled temperature (22–24 °C) and humidity (50–60%) and free access to standard rodent food and water. All animal care and experimental procedures were approved by Animal Use and Care Committee of the Korea Institute of Science and Technology (2016–035; Seoul, Korea). All experimental methods were performed in accordance with relevant guidelines and regulations.

### Cell viability

Cell viability was measured using the 3-(4,5-dimethylthiazol-2-yl)-2,5-diphenyltetrazolium bromide (MTT) assay, described elsewhere^25^. Briefly, the cells were seeded in each well of a 24-well plate and incubated in differentiation media (50 µg/ml ascorbic acid and 5 mM β-glycerophosphate) containing various concentrations of genistein (5, 10, or 20 µM). After 7 days, the cells were washed and treated with 200 µL MTT (0.5 mg/mL) for an additional 4 hours. Following additional washing, the insoluble formazan products in each well were dissolved in 200 µL dimethyl sulfoxide, and the absorbance at 550 nm of each well was recorded on a microplate spectrophotometer (Bio-Tek Instruments, Winooski, VT, USA).

### Alkaline phosphatase (ALP) assay

ALP activity was measured by enzymatic assay (Biovision, Minneapolis, MN, USA). Briefly, MC3T3-E1 cells were seeded on 24-well plates, treated with various concentrations of genistein (5, 10, or 20 µM) or transfected with siRNAs. After 3 days, the cells were washed with phosphate-buffered saline (PBS) and lysed in buffer. The cell lysates were centrifuged, and the supernatants were subjected to the assay. Absorbance of p-nitrophenol at 520 nm was determined on a microplate spectrophotometer.

### Alkaline phosphatase staining

siRNAs for five selected genes (such as *Ereg*, *Efcab2*, *Ititm5*, *Gli*, and *Hrc*) and a nonspecific control were resuspended according to the manufacturer’s instructions. siRNAs were transfected using Lipofectamine RNAiMAX (Invitrogen). A concentration of 40 pmol siRNAs was transfected into 80% confluent cells. MC3T3-E1 and M2-10B4 cells were differentiated with osteogenic media containing 5% FBS, 3 mM β-glycerophosphate and 50 μg/mL ascorbic acid in each media for 4 to 6 days. Differentiated osteoblasts were equilibrated with alkaline phosphatase (ALPL) buffer (100-mM Tris-Cl (pH 9.5), 100-mM NaCl and 10-mM MgCl2), followed by the application of 0.4 mg/mL and 0.2 mg/mL of the staining solutions nitro blue tetrazolium (NBT, Sigma) and 5-bromo-4-chloro-3-indolyl-phosphate (BCIP, Sigma), respectively. The cells were then incubated for ALPL staining at room temperature. The reaction was stopped by adding PBS containing 5-mM EDTA.

### Bone mineralization assay

The degree of bone mineralization was assessed by staining with Alizarin red S solution. MC3T3-E1 cells were treated with various concentrations of genistein (5, 10, or 20 µM) or transfected with siRNAs. After induction of differentiation for 7 days, the cells were washed with PBS and fixed with 10% formalin for 20 min. Fixed cells were stained with Alizarin red S solution (40 mM, pH 4.2) for 10 min at room temperature, and then washed four times with distilled water to remove excess stain. Alizarin red S-stained mineral deposits were dissolved in 0.1 N NaOH for quantification, and optical density was measured at 540 nm on a microplate spectrophotometer.

### RNA sequencing analysis and the identification of differentially expressed genes (DEGs)

A Trizol reagent (Invitrogen) was used to isolate total cellular RNA, and concentration and purity were determined by optical density (A260 and A260/A280, respectively) on a spectrophotometer (Bio-Tek Instruments). RNA sequencing libraries for each sample were prepared using the TruSeq RNA Library Prep Kit (Illumina, San Diego, CA, USA). Polyadenylated paired-end RNA sequencing (RNA-seq) experiments were carried out on samples prepared from three genistein-treated and three untreated samples of the mouse osteoblastic cell line MC3T3-E1. RNA-seq was performed using the Illumina HiSeq. 2000 system.

The quality of sequencing reads generated from RNA-seq experiments was checked using the FastQC program (version 0.11.5). After adaptor trimming and removal of poor-quality reads (Q <30) using Trim Galore (version 0.4.2), TopHat (tophat-2.0.10) and Bowtie (bowtie2-2.1.0) were used to map RNA-seq reads to mouse reference genome version mm10. After assembling reads to the genome using Cufflinks (cufflinks-2.2.1), gene expression was normalized against total read count (Fragment Per Kilobase of transcript per Million mapped reads, FPKM). Differentially expressed genes (DEGs) in genistein-treated cells were determined based on statistics calculated using the Cuffdiff algorithm^46^. Selection criteria for DEGs in this study were adjusted p-value (reported as q-value in Cuffdiff) <0.05 and fold change >1.5.

### Quantitative real-time reverse transcription polymerase chain reaction (qRT-PCR)

cDNA was synthesized from aliquots of 2 µg total RNA in a reaction mixture containing oligo (dT) and Reverse Transcription Premix (ELPIS-Biotech, Daejeon, Korea). qRT-PCR was performed using SYBR Green Master Mix (Roche) in the Light Cycler 480 Real-Time PCR System (Roche). Levels of mRNA encoded by specific mouse genes were determined using the specific primers listed in Supplementary Table 1.

### Western blot analysis

MC3T3-E1 cells were harvested in radio-immunoprecipitation assay lysis buffer supplemented with protease inhibitor cocktail (Sigma Aldrich). Protein concentrations were measured by Bradford assay. Western blot analysis was performed according to a standard procedure using the antibodies listed in Supplementary Table 2. Proteins were detected with the Enhanced Chemiluminescence (ECL) detection system (Amersham) and visualized by densitometry (volume of all markers/volume of β-actin, n = 3) on a LuminoImager (LAS-3000 Bio Imaging Analysis System; Fuji Film Co., Tokyo, Japan).

### siRNA treatment

siRNA transfection into MC3T3-E1 cells was performed using Lipofectamine RNAiMAX reagent (Invitrogen, New York, NY, USA). Scrambled siRNA and target gene-specific siRNAs were purchased from Santa Cruz Biotechnology (Dallas, TX, USA), Bioneer (Daejeon, Korea), and Thermo Fisher Scientific (Waltham, MA, USA). More than 60% knockdown of all targeted genes was confirmed 3 days after siRNA treatment (Figs 3B and 4B). siRNAs designed against specific mouse genes are listed in Supplementary Table 3.

### Statistical analysis

Results from qRT-PCR and western blot analyses are presented as means ± standard deviation (SD). Statistical analyses were performed using SPSS 12.0 (SPSS Inc., Chicago, IL, USA). Group differences were assessed by one-way analysis of variance (ANOVA), followed by the Duncan test. A p-value < 0.05 was considered to be statistically significant.

### Pathway and network analyses for differentially expressed genes

For biological interpretation of DEGs, DAVID (the Database for Annotation, Visualization and Integrated Discovery, https://david.ncifcrf.gov/) was employed to perform KEGG (the Kyoto Encyclopedia of Genes and Genomes) pathway analysis and Gene Ontology (GO) enrichment analysis^47,48^. Up-regulated and down-regulated genes in significant KEGG pathways (p-value < 0.05) were used to generate protein–protein interaction networks using the GeneMANIA module in Cytoscape^49^ on the basis of the mouse or human protein–protein interaction database.

## Electronic supplementary material

Supplementary Figures

Supplementary Tables
