# Author Correction: Understanding the functional role of genistein in the bone differentiation in mouse osteoblastic cell line MC3T3-E1 by RNA-seq analysis

**DOI:** 10.1038/s41598-018-24112-9

**Published:** 2018-04-18

**Authors:** Myungsuk Kim, Jisun Lim, Jung-Hee Lee, Kyung-Mi Lee, Suji Kim, Kye Won Park, Chu Won Nho, Yoon Shin Cho

**Affiliations:** 1 Convergence Research Center for Smart Farm Solution, Korea Institute of Science and Technology, Gangneung, 25451 Republic of Korea; 2 Natural Products Research Center, Korea Institute of Science and Technology, Gangneung, 25451 Republic of Korea; 3 Department of Biomedical Science, Hallym University, Chuncheon, 24252 Republic of Korea; 4 Department of Food Science and Biotechnology, Sungkyunkwan University, Suwon, 16419 Republic of Korea

Correction to: *Scientific Reports* 10.1038/s41598-018-21601-9, published online 19 February 2018

In this Article, Figure 2 is a duplication of Figure 3a and 3b. The correct Figure 2 appears below as Figure 1.

**Figure 1:**
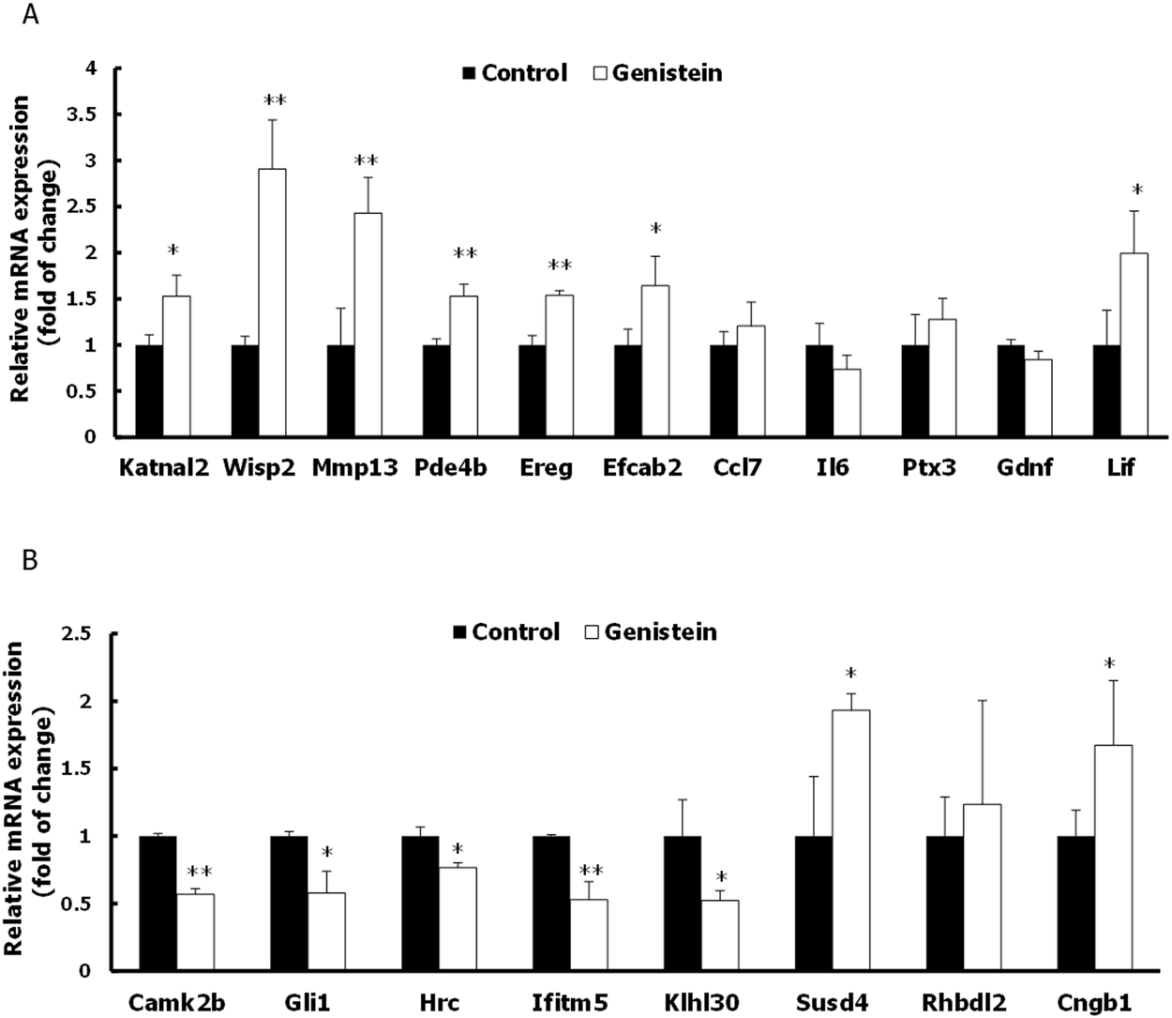
Effect of genistein on mRNA expression of selected differentially expressed genes in MC3T3-E1 cells. (**A**) Analysis of mRNA on up-regulated selected genes (*Ccl7, Lif, Mmp-13, Wisp2, Ereg, Il6, Pde4b, Katnal2, Efcab2, Gdnf*) in RNA sequencing. (**B**) Analysis of mRNA on down-regulated selected genes (*Camk2b, Cxcl9, Gli1, Hrc, Ifitm5, Klhl30, Cngb1, Rhbdl2, Susd4*) in RNA sequencing. Specific mRNA expression values were normalized to the expression of β-actin. Results are expressed as mean ± S.D of three independent experiments. (*P < 0.05, **P < 0.01 compared with control group).

