# Supplementary material for: Understanding the functional role of genistein in the bone differentiation in mouse osteoblastic cell line MC3T3-E1 by RNA-seq analysis
Source: Sci Rep. 2018 Feb 19;8:3257. doi: 10.1038/s41598-018-21601-9 (PMC5818530; doi:10.1038/s41598-018-21601-9)
Supplement: Supplementary file 1 — Supplementary Figures [file 41598_2018_21601_MOESM1_ESM.doc]

**Supplementary Notes**

**Understanding the functional role of genistein in the bone differentiation in mouse osteoblastic cell line MC3T3-E1 by RNA-seq analysis**

**Running title: RNA-seq analysis reveals the functional role of genistein in the bone differentiation**

Myungsuk Kim^1,2,§^, Jisun Lim^3,§^, Jung-Hee Lee^3^, Kyung-Mi Lee^1,2^, Suji Kim^4^, Kye Won Park^4^, Chu Won Nho^1,2,¶^, Yoon Shin Cho^3,¶^.

^1^Convergence Research Center for Smart Farm Solution, Korea Institute of Science and Technology, Gangneung, Republic of Korea.

^2^Natural Products Research Center, Korea Institute of Science and Technology, Gangneung, Republic of Korea.

^3^Department of Biomedical Science, Hallym University, Chuncheon, Gangwon-do 200-702, Republic of Korea.

^4^Department of Food Science and Biotechnology**,** Sungkyunkwan University, Suwon 16419, Republic of Korea.

^§^Myungsuk Kim and Jisun Lim equally contributed to this work.

^¶^Correspondence : Yoon Shin Cho, Professor, Department of Biomedical Science, Hallym University, Chuncheon, Gangwon-do 200-702, Republic of Korea.

Phone: 82-33-248-2111, Fax: 82-33-256-3420, E-mail: yooncho33@hallym.ac.kr (Y.S. Cho).

Chu Won Nho, Head, Convergence Research Center for Smart Farm Solution, KIST Gangneung Institute, 679 Saimdang-ro, Gangneung, Gangwon-do, Republic of Korea

Phone: 82-33-650-3651, Fax: 82-33-650-3679, E-mail: [cwnho@kist.re.kr](mailto:cwnho@kist.re.kr) (C.W. Nho).

**Supplementary Figures**

**Figure Legends**

**Supplementary Figure 1.** Differently expressed genes in between untreated- and genistein treated-mouse osteoblastic cell line MC3T3-E1 (q-value < 0.05 and fold change > 1.5)

**Supplementary Figure 2.** Effect of genistein on cell viability in MC3T3-E1 cells. Cells were grown in differentiation medium (50 μg/ml ascorbic acid and 5 mM beta-glycerophosphate) and treated with various concentration of genistein for 1 day, 3 day, or 7 day, and cell viability was determined by MTT assays. Cell viability values are expressed as percentages of vehicle controls. Results are expressed as mean ± S.D of three independent experiments. (**P* < 0.05, ***P* < 0.01 compared with scramble group)

**Supplementary Figure 3.** Effect of genistein on ALP production in MC3T3-E1 cells. ALP activity after 3 days of osteogenic induction as determined by quantitative ALP activity assay. Results are expressed as mean ± S.D of three independent experiments. (**P* < 0.05, ***P* < 0.01 compared with scramble group)

**Supplementary Figure 4.** Effect of genistein on bone mineralization in MC3T3-E1 cells. Quantification of Alizarin red S staining after 7 days of osteogenic induction. Results are expressed as mean ± S.D of three independent experiments. (**P* < 0.05, ***P* < 0.01 compared with scramble group)

**Supplementary Figure 5.** Effect of genistein on mRNA expression of selected differentially expressed genes in mouse bone marrow-derived osteoblasts. (A) Analysis of mRNA on up-regulated selected genes (*Katnal2, Wisp2, Mmp13, Pde4b, Ereg, Efcab2, Ccl7, Il6, Ptx3, Gdnf, Lif*) in RNA sequencing. (B) Analysis of mRNA on down-regulated selected genes (*Camk2b, Gli1, Hrc, Ifitm5, Klhl30, Susd4, Rhbdl2, Cngb1*) in RNA sequencing. Specific mRNA expression values were normalized to the expression of β-actin. Results are expressed as mean ± S.D of three independent experiments. (**P* < 0.05, ***P* < 0.01 compared with control group)

**Supplementary Figure 6.** ALP staining (A) and ALP activity measured by enzymatic assay (B) after 6 days of osteogenic induction for M2-10B4 cells transfected with 40 pmole siRNAs of *Ereg* and *Efcab2*.

**Supplementary Figure 7.** ALP staining (A) and ALP activity measured by enzymatic assay (B) after 6 days of osteogenic induction for M2-10B4 cells transfected with 40 pmole siRNAs of *Ifitm5*, *Gli1* and *Hrc*.

**
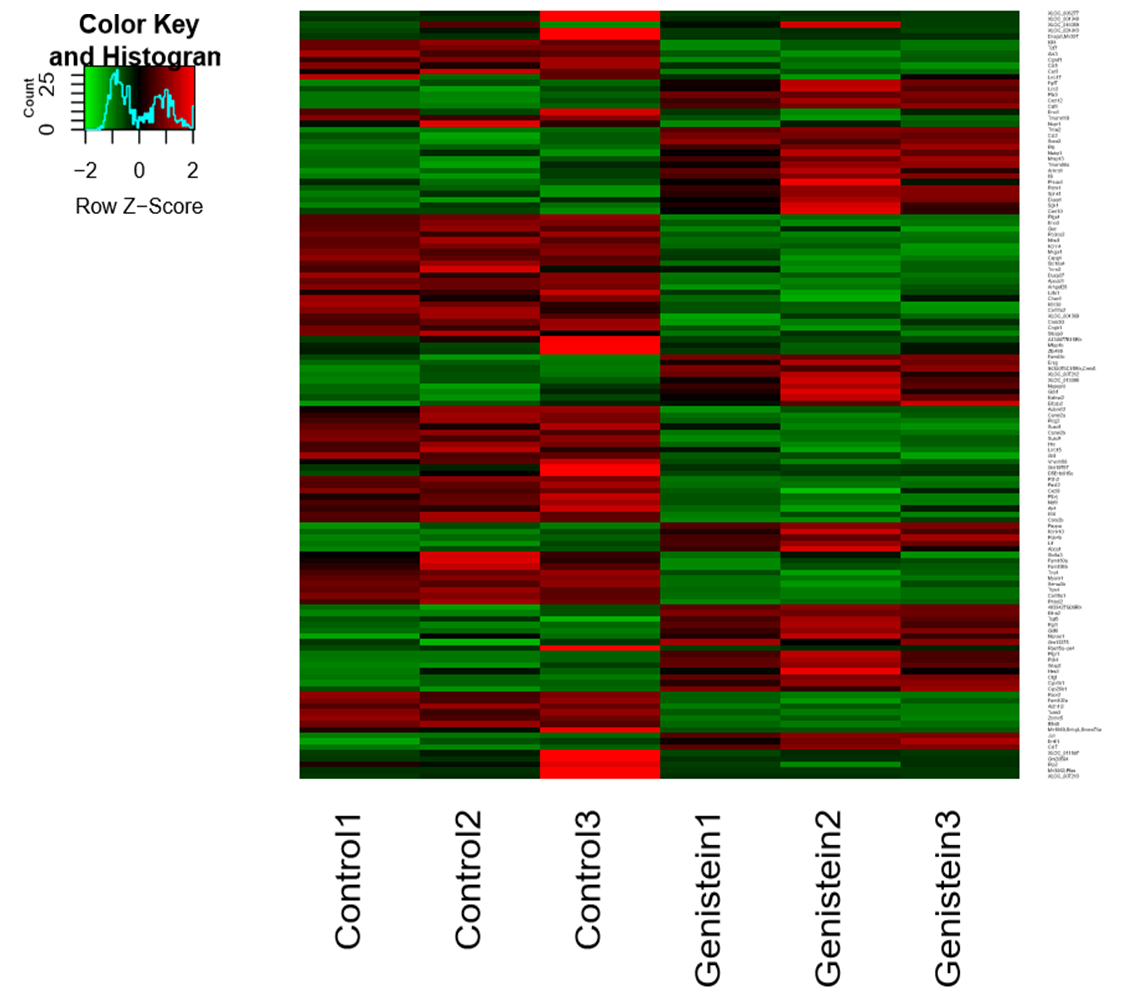
**

**Supplementary Figure 1**

**Supplementary Figure 2**

**Supplementary Figure 3**

**Supplementary Figure 4**

**Supplementary Figure 5A**

**Supplementary Figure 5B**


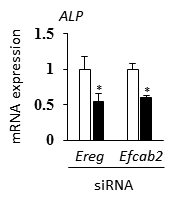
**A** **B**


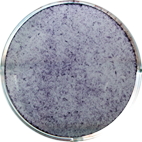

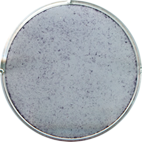


***Efcab2***

***Ereg***

**Control**

**M2-10B4**


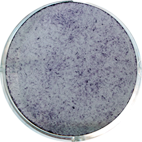


**siRNA**

**Supplementary Figure 6**

**A B**


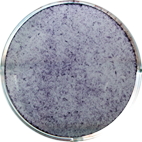

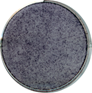

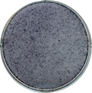

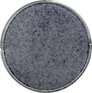


**Control**

**M2-10B4**

***Gli***

***Ifitm5***

***Hrc***

**siRNA**

**
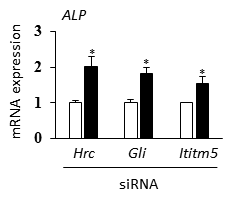
**

**Supplementary Figure 7**
